# Classification of Single Normal and Alzheimer's Disease Individuals from Cortical Sources of Resting State EEG Rhythms

**DOI:** 10.3389/fnins.2016.00047

**Published:** 2016-02-23

**Authors:** Claudio Babiloni, Antonio I. Triggiani, Roberta Lizio, Susanna Cordone, Giacomo Tattoli, Vitoantonio Bevilacqua, Andrea Soricelli, Raffaele Ferri, Flavio Nobili, Loreto Gesualdo, José C. Millán-Calenti, Ana Buján, Rosanna Tortelli, Valentina Cardinali, Maria Rosaria Barulli, Antonio Giannini, Pantaleo Spagnolo, Silvia Armenise, Grazia Buenza, Gaetano Scianatico, Giancarlo Logroscino, Giovanni B. Frisoni, Claudio del Percio

**Affiliations:** ^1^Department of Physiology and Pharmacology “Vittorio Erspamer”, University of Rome “La Sapienza”Rome, Italy; ^2^Department of Neuroscience, IRCCS San Raffaele PisanaRome, Italy; ^3^Department of Clinical and Experimental Medicine, University of FoggiaFoggia, Italy; ^4^Department of Electrical and Information Engineering, Polytechnic of BariBari, Italy; ^5^Department of Integrated Imaging, IRCCS SDN - Istituto di Ricerca Diagnostica e NucleareNapoli, Italy; ^6^Department of Motor Sciences and Healthiness, University of Naples ParthenopeNaples, Italy; ^7^Department of Neurology, IRCCS Oasi Institute for Research on Mental Retardation and Brain AgingTroina, Italy; ^8^Service of Clinical Neurophysiology (DiNOGMI; DipTeC), IRCCS Azienda Ospedaliera Universitaria San Martino - ISTGenoa, Italy; ^9^Dipartimento Emergenza e Trapianti d'Organi, University of BariBari, Italy; ^10^Gerontology Research Group, Department of Medicine, Faculty of Health Sciences, University of A CoruñaA Coruña, Spain; ^11^Department of Clinical Research in Neurology, University of Bari “Aldo Moro”, Pia Fondazione Cardinale G. PanicoLecce, Italy; ^12^Department of Basic Medical Sciences, Neurosciences and Sense Organs, University of Bari “Aldo Moro”Bari, Italy; ^13^Unit of Neurodegenerative Diseases, Department of Clinical Research in Neurology, University of Bari “Aldo Moro”, Pia Fondazione Cardinale G. PanicoLecce, Italy; ^14^Department of Imaging - Division of Radiology, Hospital “Di Venere”Bari, Italy; ^15^Division of Neuroradiology, “F. Ferrari” HospitalLecce, Italy; ^16^Department of Basic Medical Sciences, Neuroscience and Sense Organs, University of Bari “Aldo Moro”Bari, Italy; ^17^Laboratory of Epidemiology, Neuroimaging and Telemedicine, IRCCS Centro “S. Giovanni di Dio-F.B.F.”Brescia, Italy; ^18^Memory Clinic and LANVIE - Laboratory of Neuroimaging of Aging, University Hospitals and University of GenevaGeneva, Switzerland

**Keywords:** Alzheimer's disease (AD), electroencephalography (EEG), exact low-resolution brain electromagnetic tomography (eLORETA), spectral coherence, lagged linear connectivity, area under the receiver operating characteristic curve (AUROC), delta rhythms, alpha rhythms

## Abstract

Previous studies have shown abnormal power and functional connectivity of resting state electroencephalographic (EEG) rhythms in groups of Alzheimer's disease (AD) compared to healthy elderly (Nold) subjects. Here we tested the best classification rate of 120 AD patients and 100 matched Nold subjects using EEG markers based on cortical sources of power and functional connectivity of these rhythms. EEG data were recorded during resting state eyes-closed condition. Exact low-resolution brain electromagnetic tomography (eLORETA) estimated the power and functional connectivity of cortical sources in frontal, central, parietal, occipital, temporal, and limbic regions. Delta (2–4 Hz), theta (4–8 Hz), alpha 1 (8–10.5 Hz), alpha 2 (10.5–13 Hz), beta 1 (13–20 Hz), beta 2 (20–30 Hz), and gamma (30–40 Hz) were the frequency bands of interest. The classification rates of interest were those with an area under the receiver operating characteristic curve (AUROC) higher than 0.7 as a threshold for a moderate classification rate (i.e., 70%). Results showed that the following EEG markers overcame this threshold: (i) central, parietal, occipital, temporal, and limbic delta/alpha 1 current density; (ii) central, parietal, occipital temporal, and limbic delta/alpha 2 current density; (iii) frontal theta/alpha 1 current density; (iv) occipital delta/alpha 1 inter-hemispherical connectivity; (v) occipital-temporal theta/alpha 1 right and left intra-hemispherical connectivity; and (vi) parietal-limbic alpha 1 right intra-hemispherical connectivity. Occipital delta/alpha 1 current density showed the best classification rate (sensitivity of 73.3%, specificity of 78%, accuracy of 75.5%, and AUROC of 82%). These results suggest that EEG source markers can classify Nold and AD individuals with a moderate classification rate higher than 80%.

## Introduction

Alzheimer's disease (AD) is the most prevalent brain neurodegenerative disorder progressing to severe cognitive impairment and loss of autonomy (i.e., dementia) in older people (Braak and Braak, 1995; Jelic et al., 1998; Price, 2000; Leite et al., 2004; Glodzik-Sobanska et al., 2005; Kang et al., 2015).

Criteria for clinical diagnosis of AD were proposed in 1984 (McKhann et al., 1984) by the National Institute of Neurological and Communicative Disorders and Stroke (NINCDS) and by the Alzheimer's Disease and Related Disorders Association (ADRDA). According to these criteria, the final diagnosis of definite AD needed histopathologic confirmation (i.e., microscopic examination of brain tissue) in autopsy or biopsy.

In the past years, the International Working Group (IWG) and the US National Institute on Aging–Alzheimer's Association (NIA-AA) have proposed an algorithm for the diagnosis of AD at the preclinical (before any objective clinical manifestation), prodromal (with objective mild cognitive impairment [MCI] but with autonomy substantially preserved), and overt dementia stages, based on *in vivo* fluid and neuroimaging biomarkers as well as clinical phenotypes of the disease (Förstl and Kurz, 1999; Dubois et al., 2014). The neuroimaging biomarkers included brain hypometabolism, as revealed by fluorodeoxyglucose positron emission tomography (FDG-PET), brain amyloid load, as measured by ligand PET, and maps of brain atrophy and abnormalities of structural brain connectivity, as revealed by magnetic resonance imaging (MRI).

The mentioned PET and MRI methodologies capture several processes of AD, but cannot be always used due to the limited availability of the instrumental resources, costs, invasiveness, or radiation exposure (e.g., PET). These limitations are problematic especially for serial recordings over time. In contrast, electroencephalographic (EEG) recordings in awake resting state condition represent an ideal low-cost and non-invasive methodology for clinical applications. Indeed, EEG recording has a high temporal resolution (milliseconds) that provides an optimal investigational tool for the emerging features of brain physiology, namely its oscillatory nature (Schroeter et al., 2009; Babiloni et al., 2013a). Indeed, EEG rhythms are the most important features of the collective behavior of brain neural populations and are very relevant for human cognition. Furthermore, EEG procedures are widely available in any country. They are well-tolerated by patients and are not affected by the task difficulty. Moreover, they can be repeated over time without habituation effects. As an important methodological limitation, recorded EEG data require an expert manual verification of the EEG epochs free from artifacts, and commercial pieces of software do not provide immediate statistical indexes of abnormalities of EEG markers with respect to a normative database.

Previous studies in AD patients and elderly subjects with amnesic MCI have shown that resting state eyes-closed EEG rhythms may be promising markers for a neurophysiological evaluation of disease status. Noteworthy, these markers do not have a diagnostic value, as EEG rhythms do not directly reflect the pathophysiological markers of AD. Rather, they may be part of the “topographical markers” of AD according to the definition by Dubois et al. (2014). These topographic markers are not specific for AD but can provide an index of the extent to which AD subjects show the abnormal structure and/or function of the brain across time. In this framework of topographic markers, the resting state EEG rhythms may reveal abnormalities of basic neurophysiological mechanisms underlying vigilance and cognition in AD subjects. When compared to groups of normal elderly (Nold) subjects, AD groups are characterized by high power of widespread delta (< 4 Hz) and theta (4–7 Hz) rhythms, as well as by low power of posterior alpha (8–12 Hz) and/or beta (13–20 Hz) rhythms (Dierks et al., 1993, 2000; Huang et al., 2000; Ponomareva et al., 2003; Jeong, 2004). Similarly, MCI subjects display increased theta power (Grunwald et al., 2001) as well as decreased alpha power (Jelic et al., 1998, 2000; Huang et al., 2000; Grunwald et al., 2001). In line with the “transition” hypothesis, power of resting state EEG alpha rhythms is high in Nold subjects, intermediate in MCI subjects, and low in AD patients at the group level (Elmstáhl and Rosén, 1997; Huang et al., 2000; Jelic et al., 2000; Jeong, 2004).

Markers of resting state eyes-closed EEG rhythms have unveiled abnormalities of neurophysiological mechanisms in AD patients not only at the group but also at the individual level. A correct classification of up to 90% of success between Nold and AD individuals by using EEG markers as an input to artificial neural network (ANN) classifiers with the leave-one-out cross-validation method has been previously reported (Anderer et al., 1994; Pritchard et al., 1994). Similar results were obtained in the correct classification not only between AD and Nold subjects but also between Nold and MCI subjects (Adler et al., 2003; Brassen et al., 2004). Unfortunately, those results suffered from the use of a small number of subjects. Furthermore, Lehmann et al. (2007) used EEG markers as an input to random forest procedures and obtained a correct classification between AD and Nold individuals of about 82%. This classification rate improved to about 89% using ANN (Lehmann et al., 2007). Buscema and colleagues reached about 92% of success in the classification of AD and MCI individuals using EEG markers as an input to ANNs (Buscema et al., 2007), while Dauwels and colleagues reached classification rates of 83 and 88% for pre-dementia and mild AD, respectively (Dauwels et al., 2009). The same research group has also performed a recent study (Dauwels et al., 2010) comparing the classification rate between Nold and MCI individuals based on a large number of EEG markers, such as partial directed coherence, directed transfer function (DTF), full frequency DTF (ffDTF), different types of entropy measures, state space-based estimators, information-theoretic measures including Mutual Information (a measure closely related to Synchronization Likelihood), and different types of divergence and stochastic event synchrony (SES). Results revealed that most of these EEG markers globally indicated decreased functional and less effective brain connectivity in MCI patients with respect to Nold subjects. However, only two EEG markers yielded significant results in the classification of Nold and MCI individuals, namely SES and ffDTF, with a correct classification of about 83% using linear and quadratic discriminant analysis with leave-one-out cross-validation (Dauwels et al., 2010).

Previous studies have shown interesting results on markers of AD based on magnetoencephalographic (MEG) counterpart of resting state EEG rhythms. Specifically, Gómez et al. (2009) analyzed the resting state MEG rhythms in 20 AD patients and 21 Nold subjects to test the ability of the dimensional complexity of MEG signals (as measured by Higuchi's fractal dimension) to classify those individuals correctly. Results showed that MEG signals had lower complexity values in AD patients than in Nold subjects. Furthermore, the accuracy of 87.8% (80% sensitivity; 95.2% specificity) in the classification of Nold and AD subjects was obtained by computation of receiver operating characteristic (ROC) curves. Moreover, Fernández et al. (2013) demonstrated that source current density values of MEG delta rhythms in posterior parietal, occipital, pre-rolandic, and precuneus cortices distinguished reliably between MCI patients, AD patients with different severity scores, and Nold subjects. Based on these results, the Authors proposed MEG delta markers as a promising candidate for the detection of AD patients in clinical practice.

The present research group has been investigating markers of resting state eyes-closed EEG rhythms in MCI, AD, and control subjects in the framework of the “BRAINON” program (www.brainon.eu). In previous studies of this program, cortical sources of resting state eyes-closed EEG rhythms were estimated and compared between groups of MCI, AD, and control subjects, in order to enhance spatial information content of scalp-recorded EEG data and to unveil topography of EEG abnormalities associated with AD from prodromal to overt clinical stages (Babiloni et al., 2004a, 2008, 2011a,b,c, 2013a,b,c). For this purpose, low-resolution brain electromagnetic tomography (LORETA) was used (Pascual-Marqui et al., 1994). Overall, it was found that occipital, parietal, and temporal cortical sources of delta and alpha rhythms showed abnormal activity in groups of AD subjects when compared to control groups. Furthermore, this abnormal activity was related to markers of hippocampus atrophy, cortical atrophy, and vascular lesions of white matter. In the same line, when compared to control groups, groups of AD subjects showed abnormal functional coupling of these EEG rhythms as revealed by spectral coherence and other techniques of functional connectivity (Babiloni et al., 2004b, 2006b, 2008, 2009a, 2010).

As a new milestone of the “BRAINON” program, the current study tested several resting state EEG markers as ability to classify correctly AD individuals with dementia and Nold individuals. These EEG markers were obtained by the estimation of activity and functional connectivity of the cortical sources of resting state eyes-closed EEG rhythms by the use of exact LORETA (eLORETA; Pascual-Marqui, 2007a). The EEG markers of interest were used as discriminant variables for the computation of the rate of classification between single Nold and AD individuals.

## Materials and methods

### Subjects and diagnostic criteria

This study involved 120 AD and 100 Nold individuals, carefully matched for age, gender, and years of education. They were selected blindly to the features of EEG data, to avoid any logical circularity or bias in the results.

Committees of local institutional ethics approved the recording and analysis of EEG data for scientific purposes. All experiments were performed with the informed consent of each participant or caregiver, in line with the Code of Ethics of the World Medical Association (Declaration of Helsinki). The inclusion criteria for AD individuals comprised the diagnosis of mild to moderate AD according to NINCDS-ADRDA (McKhann et al., 1984) and DSM-IV guidelines. Furthermore, these individuals had to have received medical, neurological, neuropsychological, and psychiatric assessments including MMSE (Folstein et al., 1975), Clinical Dementia Rating (CDR; Hughes et al., 1982), geriatric depression scale (GDS; Yesavage et al., 1982), and Instrumental Activities of Daily Living scale (IADL; Lawton and Brody, 1969). Exclusion criteria included scores in the MMSE lower than 27 in the Nold subjects and higher than 24 in the AD subjects according to Alzheimer‘s Disease Neuroimaging Initiative, ADNI (http://adni.loni.usc.edu). Besides, exclusion criteria comprised any kind of evidence of other forms or causes of dementia such as frontotemporal dementia (The Lund Manchester Groups, 1994), vascular dementia, (NINDS-AIREN criteria; Román et al., 1993), Parkinson disease, Lewy body dementia (McKeith et al., 2005), metabolic syndrome, nutritional deficits, tumors, etc. The Nold subjects had no history of neurological or major psychiatric disorders. They underwent medical, neurological, and psychiatric assessments including MMSE and GDS, to exclude actual neurocognitive disorders and major psychiatric symptoms (including abuse of substances).

Table 1 reports information about personal and clinical characteristics of the Nold and AD subjects of the present study. An independent *t*-test evaluated the presence or absence of statistically significant differences (*p* < 0.05, one-tailed) between the two groups (i.e., Nold and AD patients) for the age, education, individual alpha frequency (IAF; see below for a description of this index), and MMSE score. As expected, a statistically significant difference was found for the MMSE score (*p* < 0.0001; higher MMSE score in the Nold than in the AD group) and for the IAF (*p* < 0.0001; higher IAF in the Nold than in the AD group). On the contrary, no statistically significant difference was found for age, gender, and education (*p* > 0.05).

**Table 1:** **Demographic and clinical data of normal elderly (Nold) subjects and Alzheimer's disease (AD) patients**.

	**Gender (Female/Male)**	**Age (years)**	**Education (years)**	**MMSE (score)**
Nold (*n* = 100)	62/38	69 ± 0.9 SE	9.7 ± 0.4 SE	28.8 ± 0.1 SE
AD (*n* = 120)	78/42	69.8 ± 0.7 SE	9.2 ± 0.4 SE	19 ± 0.3 SE

*MMSE, Mini Mental State Evaluation*.

### EEG recordings

**Figure 1:**
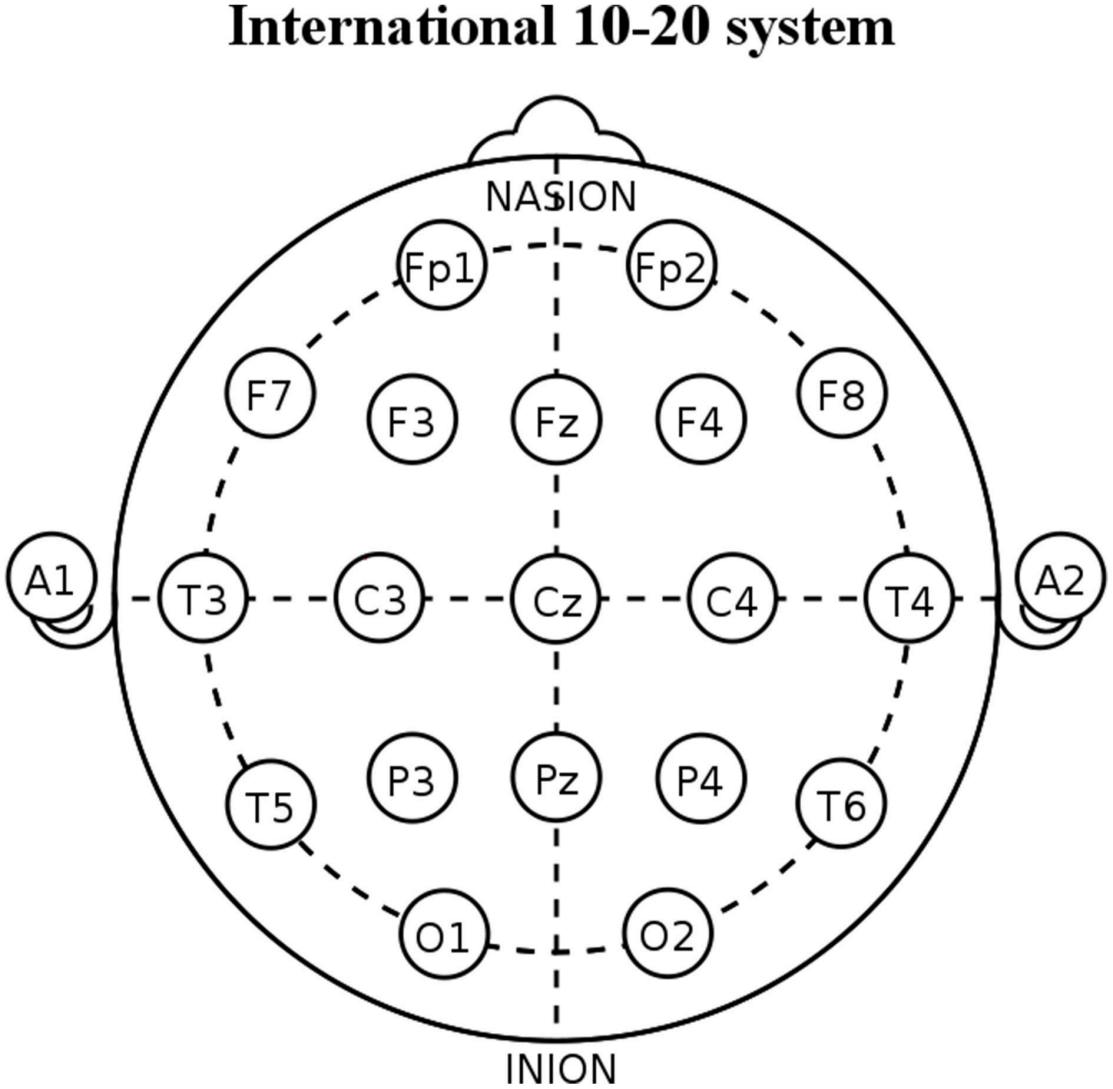
**Diagram showing the placement of the 19 scalp electrodes used for the present electroencephalographic (EEG) recordings**. These electrodes are positioned according to the International 10–20 System (i.e., Fp1, Fp2, F7, F3, Fz, F4, F8, T3, C3, Cz, C4, T4, T5, P3, Pz, P4, T6, O1, and O2). In the figure, A1 and A2 indicate the position of linked earlobe reference electrodes.

All subjects were kindly asked to stay relaxed at eyes closed and not to move or talk. EEG data were recorded (bandpass: 0.01–100 Hz; EB-Neuro Be-light©, Firenze, Italy) during resting state eyes-closed condition from 19 scalp electrodes positioned over the whole head according to the 10–20 System (i.e., Fp1, Fp2, F7, F3, Fz, F4, F8, T3, C3, Cz, C4, T4, T5, P3, Pz, P4, T6, O1, O2; Figure 1). Linked earlobe reference electrode was appreciated but not mandatory to respect the methodological facilities and standard internal protocols of the clinical recording units. A ground electrode was located between the AFz and Fz electrodes. Electrode impedance was kept below 5 Kohm. All recorded artifact-free EEG data were off-line re-referenced to common average to harmonize the EEG data collected with different reference electrodes. The horizontal and vertical electro-oculographic activities (0.3–70 Hz bandpass) were simultaneously recorded to monitor eye movements. All data were digitalized in continuous recording mode (about 5 min of EEG; 128–256 Hz sampling rate set to avoid aliasing).

### Preliminary EEG data analysis

The EEG data were segmented in consecutive epochs of 2 s and were analyzed off-line. The epochs affected by any artifacts (ocular, muscular, instrumental) were preliminarily identified by an automatic computerized procedure. Two independent experimenters manually confirmed the artifact-free EEG epochs that were accepted for further analysis. Particular attention was paid to exclude EEG epochs with signs of drowsiness or pre-sleep stages.

### Spectral analysis of EEG rhythms

The power spectral density of the EEG rhythms was computed using an FFT analysis (Welch method, Hanning windowing function, no phase shift) with 0.5 Hz of frequency resolution. The following standard frequency bands of interest were considered, in line with previous relevant EEG studies (Besthorn et al., 1997; Chiaramonti et al., 1997; Babiloni et al., 2005, 2006b, 2011b, 2013c): delta (2–4 Hz), theta (4–8 Hz), alpha 1 (8–10.5 Hz), alpha 2 (10.5–13 Hz), beta 1 (13–20 Hz), beta 2 (20–30 Hz), and gamma (30–40 Hz). These frequency bands were computed sharing the frequency bin by two contiguous bands, as a widely accepted procedure (Jelic et al., 1996; Besthorn et al., 1997) that fits the theoretical consideration that near EEG rhythms may overlap at their frequency borders (Klimesch et al., 1996; Klimesch, 1999; Babiloni et al., 2005, 2010). Individual alpha frequency (IAF) peak was defined as the frequency associated with the strongest EEG power at the extended alpha range of 7–14 Hz (Klimesch, 1999).

### Cortical source of EEG rhythms as computed by eLORETA

As mentioned above, eLORETA estimated the activity of the cortical sources of EEG rhythms (Pascual-Marqui, 2007a). eLORETA is an improvement over previous well-known techniques called LORETA (Pascual-Marqui et al., 1994) and the standardized LORETA (sLORETA; Pascual-Marqui, 2002). In simulation studies, both sLORETA and eLORETA showed low spatial resolution but zero localization error in the presence of measurement and biological noise (Pascual-Marqui, 2002, 2007a). Furthermore, it was reported a better source location by eLORETA than sLORETA (Canuet et al., 2011).

eLORETA uses a realistic head model (Fuchs et al., 2002) using the MNI152 template (Mazziotta et al., 2001), with a three-dimensional solution space restricted to cortical gray matter and electrode coordinates provided by Jurcak (Jurcak et al., 2007). The intracerebral volume is partitioned in 6239 voxels with 5 mm spatial resolution. Thus, eLORETA images obtained from resting state EEG data represent the electrical activity at each voxel in the neuroanatomic Montreal Neurological Institute (MNI) space as the exact magnitude of the estimate current density. Anatomical labels as Brodmann areas are also reported using MNI space, with adaptation to Talairach space (Brett et al., 2002).

eLORETA cortical source solutions did estimate current density values at x, y, and z vectors of any brain voxel able to predict EEG spectral power density at all scalp electrodes. Among the infinite solutions to the EEG inverse problem, a regularization procedure selected the maximally smoothed solution at the cortical source level of the eLORETA head model. Afterwards, this solution was normalized by the computation of the eLORETA current density at each voxel (as the mean of the x, y, and z vectors) with current density value averaged across all frequencies (0.5–45 Hz) and 6239 voxels of the brain volume. For this reason, normalized eLORETA solutions are reported by an arbitrary unit scale in which “1” means equal to the average value of eLORETA current density computed across all frequencies and voxels. The general procedure typical fits EEG power density in a Gaussian distribution and reduces inter-subject variability (Leuchter et al., 1993).

In line with the general low spatial resolution of the present EEG methodological approach (i.e., 19 scalp electrodes), the eLORETA solutions were averaged across all voxels in a given cortical macro region of interest (ROI). Frontal, central, parietal, occipital, temporal, and limbic ROIs were considered. Table 2 reports the Brodmann areas (BAs) included in these ROIs. The eLORETA cortical sources at these ROIs and all frequency bands of interest were used as a first set of EEG markers for the present classification purposes.

**Table 2:** **Regions of interest (ROIs) for the estimation of the cortical sources of resting state eyes-closed electroencephalographic (EEG) rhythms by exact low-resolution brain electromagnetic tomography (eLORETA) software**.

Brodmann areas into the regions of interest (ROIs)	
Frontal	8, 9, 10, 11, 44, 45, 46, 47
Central	1, 2, 3, 4, 6
Parietal	5, 7, 30, 39, 40, 43
Temporal	20, 21, 22, 37, 38, 41, 42
Occipital	17, 18, 19
Limbic	31, 32, 33, 34, 35, 36

*Any ROI is defined by some Brodmann areas within the cortical source space of the eLORETA software*.

### EEG lagged linear connectivity by eloreta

Functional connectivity between two regions has previously been defined as the non-linear and linear dependence, as for example, lagged non-linear and linear coherence of intra-cortical EEG-source estimates (Pascual-Marqui, 2007b). When computed in the cortical source space, the inherent low spatial resolution of the EEG tomography enters high phase synchronization and zero-lag coherence (Pascual-Marqui, 2007b). Activity at any cortical area is observed instantaneously (zero-lag) by all scalp electrodes. Instantaneous coherence between two cortical sources might also be found when a third source has an impact on two other brain sources even whether the two paired sources do not influence each other or as there is activity at reference electrode in coherence analysis. For this reason, only the lagged coherence contribution of these measures should be considered for more save neurophysiological considerations. In this line, Pascual-Marqui (2007b) proposed the solution to remove the zero-lag instantaneous interactions and to compute coherence using the residual, corrected time series. Furthermore, the proposed solution included measures of dependence among multivariate EEG time series (Pascual-Marqui, 2007c). This procedure of functional connectivity was called lagged linear connectivity (LLC) and was implemented to make it available a freeware in the eLORETA package (Pascual-Marqui et al., 2011). In the present study, we used that freeware (Pascual-Marqui et al., 2011) to test the classification accuracy of EEG markers of functional connectivity among EEG cortical sources in Nold and AD individuals.

For each subject and frequency band of interest (i.e., delta, theta, alpha 1, alpha 2, beta 1, beta 2, and gamma), the LLC was computed for six ROIs (i.e., frontal, central, parietal, occipital, temporal, and limbic). For the inter-hemispherical analysis, the LLC estimates were calculated between all voxels of the mentioned ROIs of each hemisphere with the corresponding ones of the other hemisphere. The LLC solutions for all voxels of a given pair of ROIs were averaged. For the intra-hemispherical analysis, the LLC estimates were computed for all voxels of a particular ROI with all voxels of another ROI of the same hemisphere. The LLC solutions for all voxels of a given pair of ROIs were averaged. This operation was repeated for the left/and the right hemisphere.

### Statistical analysis of the eLORETA computations

To test the working hypotheses related to EEG power activity and functional connectivity as revealed by eLORETA solutions, two statistical sessions were performed by the commercial tool STATISTICA 10 (StatSoft Inc., www.statsoft.com).

The first statistical session tested the hypothesis that mean current density in the ROIs would differ between the Nold and AD groups. To this aim, a Three-way ANOVA was computed using regional normalized eLORETA solutions (normalized current density at all voxels of a given ROI) as a dependent variable (*p* < 0.05). The ANOVA factors were Group (Nold, AD), Band (delta, theta, alpha 1, alpha 2, beta 1, beta 2, and gamma), and ROI (frontal, central, parietal, occipital, temporal, and limbic). Subjects' age, education, IAF, and gender were used as covariates. Mauchly's test evaluated the sphericity assumption. The degrees of freedom were corrected by Greenhouse-Geisser procedure when appropriate. Duncan test was used for *post-hoc* comparisons (*p* < 0.05, one-tailed). Of note, the present study was not focused on the differences of EEG cortical sources between the Nold and the AD group. That focus would have required a conservative *post-hoc* test using a correction for multiple univariate comparisons at *p* < 0.05. Rather, this study tested the ability of EEG source markers to classify Nold and AD individuals. In this line, ANOVA and *post-hoc* tests were used merely to reduce the amount of EEG markers to be used for the calculation of that ability. For this reason, we used the liberal one-tailed analysis rather than a conservative correction for multiple univariate statistical comparisons.

The second statistical session tested the hypothesis that the mean functional connectivity among ROIs would differ between the Nold and AD groups. Different Three-way ANOVAs were performed using the LLC calculated with eLORETA as a dependent variable (*p* < 0.05). The first ANOVA tested the mean differences of inter-hemispherical LLC between the two groups. The ANOVA factors were Group (Nold, AD), Band (delta, theta, alpha 1, alpha 2, beta 1, beta 2, and gamma), and ROI pairs (frontal left-frontal right, central left-central right, parietal left-parietal right, occipital left-occipital right, temporal left-temporal right, and limbic left-limbic right). The second ANOVA tested the mean differences of left intra-hemispherical LLC between the two groups. The ANOVA factors were Group (Nold, AD), Band (delta, theta, alpha 1, alpha 2, beta 1, beta 2, and gamma), and ROI pairs (frontal-central, frontal-parietal, frontal-occipital, frontal-temporal, frontal-limbic, central-parietal, central-occipital, central, temporal, central-limbic, parietal-occipital, parietal-temporal, parietal-limbic, occipital-temporal, occipital-limbic, and temporal-limbic). The third ANOVA had the same design for the right hemisphere. In the three ANOVAs, subjects' age, education, IAF, and gender were used as covariates. Mauchly's test evaluated the sphericity assumption. The degrees of freedom were corrected by Greenhouse-Geisser procedure when appropriate. Duncan test was used for *post-hoc* comparisons (*p* < 0.05, one-tailed).

### Accuracy of the EEG markers in the discrimination between Nold and AD individuals

The third statistical session tested the ability of the EEG markers (i.e., source current density and LLC) to classify single Nold and AD individuals. To this aim, the EEG markers showing statistical differences between the Nold and the AD group in those two statistical sessions were used as input variables for the third statistical session. The classification rate was computed by the analysis of the receiver operating characteristic curve (ROC; DeLong et al., 1988). This analysis was performed by Matlab 2010b software (Mathworks Inc., Natick, MA, USA). The following indexes measured the classification rate of the above binary classification (i.e., Nold vs. AD):

1. Sensitivity defined as the rate of the AD subjects correctly classified as AD (i.e., true positive rate);
2. Specificity defined as the rate of the Nold (control) subjects correctly classified as Nold (i.e., true negative rate);
3. Accuracy defined as the mean between the sensitivity and specificity;
4. The area under the ROC curve (AUROC) expressed as a percentage. Noteworthy, the EEG markers of interest were those showing an AUROC higher than an arbitrary threshold of 70% (i.e., the threshold of the so-called “moderate” classification rate, DeLong et al., 1988).

### Evaluation of the relationship between the EEG markers and relevant variables of AD

Based on the values of the EEG markers, we defined AD individuals with the EEG marker “positive” (i.e., AD+) as those having marker values equal or higher than the mean plus one standard deviation (SD) of the marker value in the Nold reference population. This AD+ condition is expected to indicate abnormal values of the EEG marker in AD individuals as compared to the Nold group. In this line, the AD individuals with the EEG marker “negative” (i.e., AD−) were those having marker values within the mean plus one SD of the marker value in the Nold reference population. To test the hypothesis that the AD+ condition is related to relevant disease variables in AD patients, the MMSE score was compared between the AD+ subgroup and the AD− subgroup by independent *t*-test (*p* < 0.05, one tailed).

In the same line, we also hypothesized that the AD+ condition is related to more abnormal brain structure in AD patients. To test this hypothesis, cortical gray matter (GM), subcortical white matter (WM), and cerebrospinal fluid (CSF) normalized volumes were estimated in 39 of the 120 AD patients (those having T1- and T2-weigthed structural magnetic resonance imaging-MRI available). These MRIs had been acquired following standard research settings mostly by 1.5 T scanners. The MRI scans were visually inspected to verify the absence of structural abnormalities or technical artifacts. Centralized MRI data analysis was performed by MATLAB 7.1 (MathWorks, Natick, MA) and SPM8 (Wellcome Dept. Cogn. Neurol., London; http://www.fil.ion.ucl.ac.uk/spm).

Specifically, the processing of the MRI data was as follows: the native MRI data of each patient were partitioned into GM, WM, and CSF compartments and spatially normalized to fit a standard labeled template, obtaining the transformation matrix (Ashburner and Friston, 1997). This labeled template was based on averaged high-resolution MRIs acquired from 24 subjects, comprising anatomic channels (T1, T2, and proton density weighted), tissue channels (CSF probability, GM probability, WM probability, and tissue labels), and the LPBA40 cortical parcellation map, based on the LONI Probabilistic Brain Atlas of 40 subjects (Shattuck et al., 2008) and identifying 56 brain structures. Second, the inverse of the transformation matrix was used to map the 56 atlas brain structures to the partitioned GM compartment, enabling volume quantification of each structure. Thirdly, a homemade MATLAB script was used to calculate the volume of cortical GM, and of the entire (cortical and subcortical) GM, WM, and CSF compartments. The normalized volumes of cortical GM, and WM and CSF compartments were obtained dividing the volume of each compartment by the total (GM, WM, and CSF) volume. Of note, the above procedure seemed to be more appropriate than voxel-based morphometry (Good et al., 2001) for the analysis of the relationship between low-resolution (LORETA) EEG source estimates and MRI markers. Indeed, voxel-based morphometry is based on an intrinsically high-resolution voxel-by-voxel approach.

The comparison of the GM, WM, and CSF normalized volumes in the AD+ subgroup and in the AD− subgroup was performed by a Two-way ANOVA using the normalized volume as a dependent variable (*p* < 0.05). The ANOVA factors were Group (AD+, AD−) and Brain volume (GM, WM, and CSF). Mauchly's test evaluated the sphericity assumption. Correction of the degrees of freedom was made with Greenhouse-Geisser procedure when appropriate. The Duncan test was used for *post-hoc* comparisons (*p* < 0.05, one tailed).

All the analysis performed to test the relationship between the EEG markers and relevant variables of AD (MMSE scores, GM, WM, and CSF normalized volumes) constituted the fourth statistical session.

## Results

### Results of the first statistical session: Source current density of EEG rhythms

**Figure 2:**
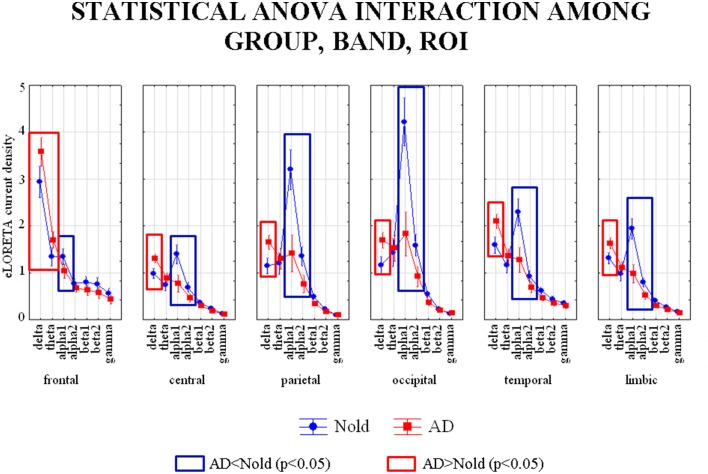
**Diagram showing the grand average of regional normalized exact low-resolution brain electromagnetic tomography (eLORETA) solutions (i.e., source activity) relative to a statistically significant ANOVA interaction [*F*_(30, 6540)_ = 18.727, *p* < 0.0001] among the factors Group (AD, Nold), Band (delta, theta, alpha 1, alpha 2, beta 1, beta 2, gamma), and ROI (central, frontal, parietal, occipital, temporal, limbic)**. Subjects' age, education, IAF, and gender were used as covariates. Legend: the rectangles indicate the cortical regions and frequency bands in which source activity presented the statistically significant LORETA pattern of source activity Nold ≠ AD (Duncan test, *p* < 0.05).

Figure 2 shows the grand average across subjects of the activity of regional EEG cortical sources relative to a statistically significant ANOVA interaction [*F*_(30, 6540)_ = 18.727, *p* < 0.0001] among the factors Group (Nold, AD), ROI (frontal, central, parietal, occipital, temporal, and limbic), and Band (delta, theta, alpha 1, alpha 2, beta 1, beta 2, gamma). Subjects' age, education, IAF, and gender were used as covariates. Planned *post-hoc* testing unveiled the following statistically significant results (see Table 3). Compared to the Nold group, the AD group showed a higher delta current density in frontal, central, parietal, occipital, temporal, and limbic regions, as well as a higher theta current density in the frontal region. The AD group also showed a lower alpha 1 current density in frontal, central, parietal, occipital, temporal, and limbic regions, as well as a lower alpha 2 current density in central, parietal, occipital, temporal, and limbic regions.

**Table 3:** ***P*-values (Duncan *post-hoc*) of the ANOVA related to the comparisons of source activity showing a statistically significant interaction [*F*_(30, 6540)_ = 18.727, *p* < 0.0001] among the factors Group (AD, Nold), Band (delta, theta, alpha 1, alpha 2, beta 1, beta 2, gamma), and ROI (central, frontal, parietal, occipital, temporal, limbic) and *p*-values of the *t*-tests for each composite EEG marker**.

***p*-values of the ANOVA and *t*-test**						
	**Frontal**	**Central**	**Parietal**	**Occipital**	**Temporal**	**Limbic**
Delta	0.00001	0.001	0.000002	0.000002	0.000005	0.001
Theta	0.0004	n.s.	n.s.	n.s.	n.s.	n.s.
Alpha1	0.002	0.000001	0.000002	0.000005	0.000002	0.000001
Alpha2	n.s.	0.03	0.000001	0.000001	0.02	0.01
Delta/Alpha1	0.00004	0.000004	0.0000001	0.0000001	0.0000001	0.000001
Theta/Alpha1	0.0000001	–	–	–	–	–
Delta/Alpha2	–	0.00007	0.0000001	0.0000001	0.0000001	0.0000001

### Results of the second statistical session: Lagged linear connectivity (LLC)

**Figure 3:**
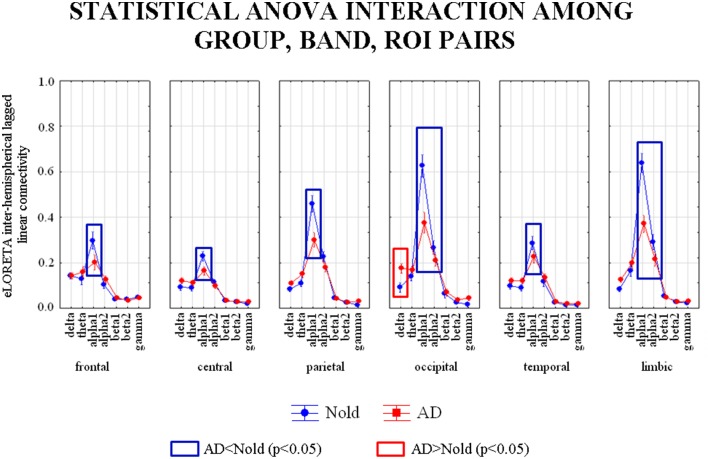
**Diagram showing the grand average of the EEG inter-hemispherical lagged linear connectivity computed between left and right hemispheres in the six regions of interest (ROI)**. These values refer a statistically significant ANOVA interaction [*F*_(30, 6540)_ = 4.8771, *p* < 0.0001] among the factors Group (Nold, and MCI), Band (delta, theta, alpha 1, alpha 2, beta 1, beta 2, gamma), and ROI pairs (frontal left-frontal right, central left-central right, parietal left-parietal right, occipital left-occipital right, temporal left-temporal right, and limbic left-limbic right). Subjects' age, education, IAF, and gender were used as covariates. Legend: the rectangles indicate the ROIs and frequency bands in which connectivity values presented the pattern Nold ≠ AD (Duncan test, *p* < 0.05).

Figure 3 shows the grand average across subjects of the inter-hemispherical LLC values relative to a statistically significant ANOVA interaction [*F*_(30, 6540)_ = 4.8771, *p* < 0.0001] among the factors Group (AD, Nold), ROI pairs (frontal left-frontal right, central left-central right, parietal left-parietal right, occipital left-occipital right, temporal left-temporal right, limbic left-limbic right), and Band (delta, theta, alpha 1, alpha 2, beta 1, beta 2, gamma). Subjects' age, education, IAF, and gender were used as covariates. Planned *post-hoc* testing exhibited the following results (see Table 4). Compared to the Nold group, the AD group showed higher delta inter-hemispherical LLC at occipital region, lower alpha 1 inter-hemispherical LLC at frontal, central, parietal, occipital, temporal, and limbic regions, as well as a lower alpha 2 inter-hemispherical LLC at occipital and limbic regions.

**Table 4:** ***p*-values (Duncan *post-hoc*) of the ANOVA related to the comparisons of inter-hemispherical lagged linear connectivity showing a statistically significant interaction [*F*_(30, 6540)_ = 4.8771, *p* < 0.0001] among the factors Group (AD, Nold), ROI pairs (frontal left-frontal right, central left-central right, parietal left-parietal right, occipital left-occipital right, temporal left-temporal right, limbic left-limbic right), and Band (delta, theta, alpha 1, alpha 2, beta 1, beta 2, gamma) and *p*-values of the *t*-tests for each composite EEG marker**.

***p*-values of the ANOVA and *t*-test**						
	**Frontal**	**Central**	**Parietal**	**Occipital**	**Temporal**	**Limbic**
Delta	n.s.	n.s.	n.s.	0.0007	n.s.	n.s.
Theta	n.s.	n.s.	n.s.	n.s.	n.s.	n.s.
Alpha1	0.00003	0.01	0.000003	0.00001	0.008	0.000001
Alpha2	n.s.	n.s.	n.s.	0.01	n.s.	0.001
Delta/Alpha1	–	–	–	0.03	–	–
Delta/Alpha2	–	–	–	n.s.	–	–

**Figure 4:**
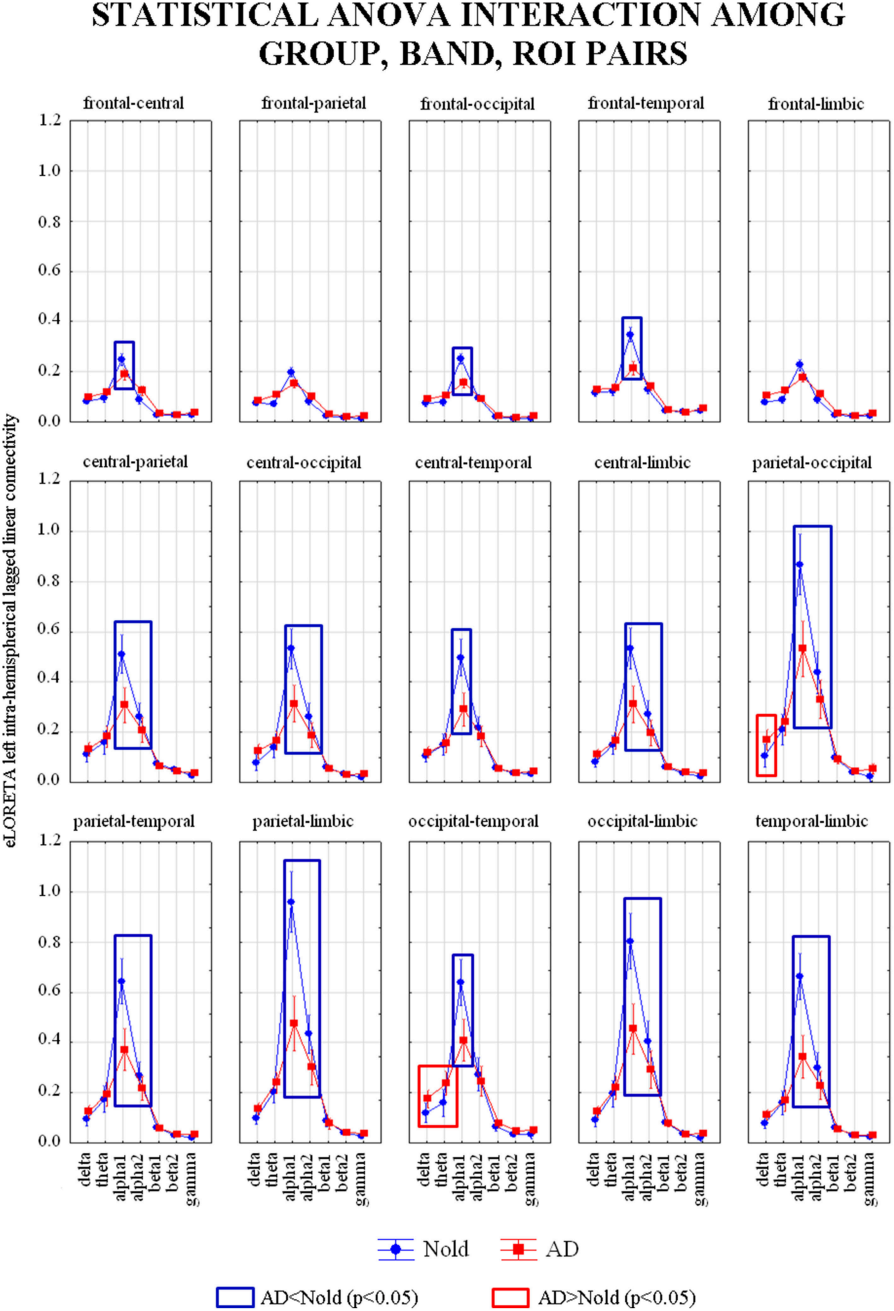
**Diagram showing the grand average of the EEG intra-hemispherical lagged linear connectivity computed between each pair of regions of interest (ROI) in the left hemisphere**. These values refer a statistically significant ANOVA interaction [*F*_(84, 18312)_ = 8.5942, *p* < 0.0001] among the factors Group (AD, Nold), ROI pairs (frontal-central, frontal-parietal, frontal-occipital, frontal-temporal, frontal-limbic, central-parietal, central-occipital, central, temporal, central-limbic, parietal-occipital, parietal-temporal, parietal-limbic, occipital-temporal, occipital-limbic, and temporal-limbic), and Band (delta, theta, alpha 1, alpha 2, beta 1, beta 2, gamma). Subjects' age, education, IAF, and gender were used as covariates. Legend: the rectangles indicate the ROI pairs and frequency bands in which connectivity values presented the pattern Nold ≠ AD (Duncan test, *p* < 0.05).

Figure 4 shows the grand average across subjects of the left intra-hemispherical LLC values relative to a statistically significant ANOVA interaction [*F*_(84, 18312)_ = 8.5942, *p* < 0.0001] among the factors Group (AD, Nold), ROI pairs (frontal-central, frontal-parietal, frontal-occipital, frontal-temporal, frontal-limbic, central-parietal, central-occipital, central-temporal, central-limbic, parietal-occipital, parietal-temporal, parietal-limbic, occipital-temporal, occipital-limbic, and temporal-limbic), and Band (delta, theta, alpha 1, alpha 2, beta 1, beta 2, gamma). Subjects' age, education, IAF, and gender were used as covariates. Planned *post-hoc* testing exhibited the following results (see Table 5). Compared to the Nold group, the AD group showed higher delta left intra-hemispherical LLC at parietal-occipital and occipital-temporal pairs as well as higher theta left intra-hemispherical LLC at occipital-temporal pair. Besides, the AD group showed lower alpha 1 left intra-hemispherical LLC at frontal-central, frontal-occipital, frontal-temporal, central-parietal, central-occipital, central-temporal, central-limbic, parietal-occipital, parietal-temporal, parietal-limbic, occipital-temporal, occipital-limbic, and temporal-limbic pairs as well as a lower alpha 2 left intra-hemispherical LLC at central-parietal, central-occipital, central-limbic, parietal-occipital, parietal-temporal, parietal-limbic, occipital-limbic, and temporal-limbic pairs.

**Table 5:** ***p*-values (Duncan *post-hoc*) of the ANOVA related to the comparisons of left intra-hemispherical lagged linear connectivity showing a statistically significant interaction [*F*_(84, 18312)_ = 8.5942, *p* < 0.0001] among the factors Group (AD, Nold), ROI pairs (frontal-central, frontal-parietal, frontal-occipital, frontal-temporal, frontal-limbic, central-parietal, central-occipital, central-temporal, central-limbic, parietal-occipital, parietal-temporal, parietal-limbic, occipital-temporal, occipital-limbic, and temporal-limbic), and Band (delta, theta, alpha 1, alpha 2, beta 1, beta 2, gamma) and *p*-values of the *t*-tests for each composite EEG marker**.

	***p*-values of the ANOVA and *t*-test**				
	**Delta**	**Theta**	**Alpha1**	**Alpha2**	**Delta/Alpha1**
Frontal-central	n.s.	n.s.	0.02	n.s.	–
Frontal-parietal	n.s.	n.s.	n.s.	n.s.	–
Frontal-occipital	n.s.	n.s.	0.00008	n.s.	–
Frontal-temporal	n.s.	n.s.	0.000001	n.s.	–
Frontal-limbic	n.s.	n.s.	n.s.	n.s.	–
Central–parietal	n.s.	n.s.	0.000002	0.03	–
Central-occipital	n.s.	n.s.	0.000002	n.s.	–
Central-temporal	n.s.	n.s.	0.000002	n.s.	–
Central-limbic	n.s.	n.s.	0.000002	0.03	–
Parietal-occipital	0.01	n.s.	0.000005	0.000006	0.02
Parietal-temporal	n.s.	n.s.	0.000002	0.03	–
Parietal-limbic	n.s.	n.s.	0.000002	0.000001	–
Occipital-temporal	0.01	0.002	0.000001	n.s.	0.02
Occipital-limbic	n.s.	n.s.	0.000001	0.000002	–
Temporal-limbic	n.s.	n.s.	0.000002	0.002	–

**Figure 5:**
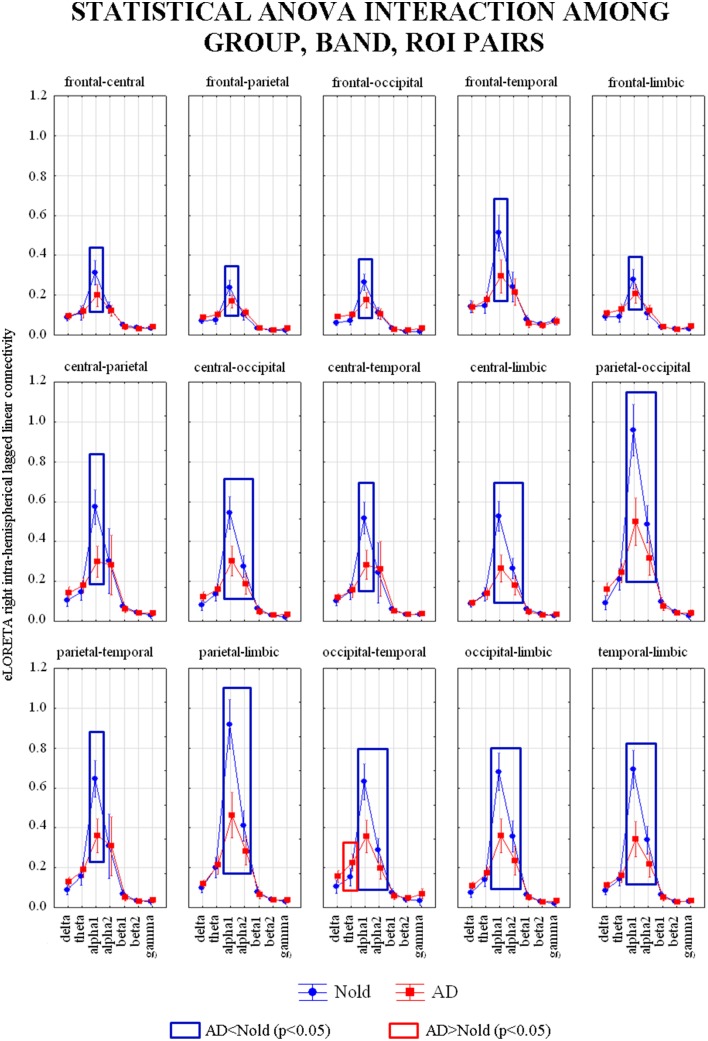
**Diagram showing the grand average of the EEG intra-hemispherical lagged linear connectivity computed between each pair of regions of interest (ROI) in the right hemisphere**. These values refer a statistically significant ANOVA interaction [*F*_(84, 18312)_ = 4.6296, *p* < 0.0001] among the factors Group (AD, Nold), ROI pairs (frontal-central, frontal-parietal, frontal-occipital, frontal-temporal, frontal-limbic, central-parietal, central-occipital, central-temporal, central-limbic, parietal-occipital, parietal-temporal, parietal-limbic, occipital-temporal, occipital-limbic, and temporal-limbic), and Band (delta, theta, alpha 1, alpha 2, beta 1, beta 2, gamma). Subjects' age, education, IAF, and gender were used as covariates. Legend: the rectangles indicate the ROI pairs and frequency bands in which connectivity values presented the pattern Nold ≠ AD (Duncan test, *p* < 0.05).

Figure 5 shows the grand average across subjects of the right intra-hemispherical LLC values relative to a statistically significant ANOVA interaction [*F*_(84, 18312)_ = 4.6296, *p* < 0.0001] among the factors Group (AD, Nold), ROI pairs (frontal-central, frontal-parietal, frontal-occipital, frontal-temporal, frontal-limbic, central-parietal, central-occipital, central-temporal, central-limbic, parietal-occipital, parietal-temporal, parietal-limbic, occipital-temporal, occipital-limbic, and temporal-limbic), and Band (delta, theta, alpha 1, alpha 2, beta 1, beta 2, gamma). Subjects' age, education, IAF, and gender were used as covariates. Planned *post-hoc* testing exhibited the following results (see Table 6). Compared to the Nold group, the AD group showed higher theta right intra-hemispherical LLC at occipital-temporal pair. Besides, the AD group showed lower alpha 1 right intra-hemispherical LLC at all the ROI pairs, as well as a lower alpha 2 right intra-hemispherical LLC at central-occipital, central-limbic, parietal-occipital, parietal-limbic, occipital-temporal, occipital-limbic, and temporal-limbic pairs.

**Table 6:** ***p*-values (Duncan *post-hoc*) of the ANOVA related to the comparisons of right intra-hemispherical lagged linear connectivity showing a statistically significant interaction interaction [*F*_(84, 18312)_ = 4.6296, *p* < 0.0001] among the factors Group (AD, Nold), ROI pairs (frontal-central, frontal-parietal, frontal-occipital, frontal-temporal, frontal-limbic, central-parietal, central-occipital, central-temporal, central-limbic, parietal-occipital, parietal-temporal, parietal-limbic, occipital-temporal, occipital-limbic, and temporal-limbic), and Band (delta, theta, alpha 1, alpha 2, beta 1, beta 2, gamma) and *p*-values of the *t*-tests for each composite EEG marker**.

***p*-values of the ANOVA and *t*-test**						
	**Delta**	**Theta**	**Alpha1**	**Alpha2**	**Theta/Alpha1**	**Theta/Alpha2**
Frontal-central	n.s.	n.s.	0.0004	n.s	–	–
Frontal-parietal	n.s.	n.s.	0.04	n.s.	–	–
Frontal-occipital	n.s.	n.s.	0.006	n.s.	–	–
Frontal-temporal	n.s.	n.s.	0.000002	n.s.	–	–
Frontal-limbic	n.s.	n.s.	0.02	n.s.	–	–
Central-parietal	n.s.	n.s.	0.000001	n.s.	–	–
Central-occipital	n.s.	n.s.	0.000001	0.007	–	–
Central-temporal	n.s.	n.s.	0.000001	n.s.	–	–
Central-limbic	n.s.	n.s.	0.000001	0.03	–	–
Parietal-occipital	n.s.	n.s.	0.000002	0.000001	–	–
Parietal-temporal	n.s.	n.s.	0.000002	n.s.	–	–
Parietal-limbic	n.s.	n.s.	0.000002	0.00003	–	–
Occipital-temporal	n.s.	n.s.	0.000002	0.007	0.00001	0.02
Occipital–limbic	n.s.	n.s.	0.000002	0.000008	–	–
Temporal–limbic	n.s.	n.s.	0.000002	0.0007	–	–

### Results of the third statistical session: Accuracy of the EEG markers

The results of the first statistical session showed that compared to the Nold group, the AD group was characterized by higher delta source activity and lower alpha 1 source activity in several cortical regions, as well as higher theta activity in frontal region. Therefore, we decided to use composite EEG markers of source activity besides the simple EEG markers. This decision allowed an integration of the information content conveyed by the EEG markers of current density. Specifically, the composite EEG markers were obtained by computing the ratio between the source activities of the different EEG ryhthms when these activities were statistically abnormal in the same cortical region of interest. In addition, an independent *t*-test analysis were performed for each composite EEG marker (see Table 3), in order to ensure that the statistical differences continued being significant for the composite markers. Those composite EEG markers which not showed significant differences between the groups were not included as discriminant variables for the analysis of the ROC curves.

In total, the following composite EEG markers of current density were formed: (1) delta/alpha 1 current density in frontal region; (2) delta/alpha 1 current density in central region; (3) delta/alpha 1 current density in parietal region; (4) delta/alpha 1 current density in occipital region; (5) delta/alpha 1 current density in temporal region; (6) delta/alpha 1 current density in limbic region; (7) theta/alpha 1 current density in frontal region; (8) delta/alpha 2 current density in central region; (9) delta/alpha 2 current density in parietal region; (10) delta/alpha 2 current density in occipital region; (11) delta/alpha 2 current density in temporal region; (12) delta/alpha 2 current density in limbic region

The results of the second statistical session also showed that compared to the Nold group, the AD group was characterized by abnormal inter- and intra-hemispherical LLC in several cortical regions. Therefore, the same procedure was followed to form composite EEG markers of connectivity (see *p*-values for the *t*-tests in Tables 4–6). The following composite EEG markers of LLC were formed: (1) delta/alpha 1 inter-hemispherical LLC in occipital region; (2) delta/alpha 1 left intra-hemispherical LLC in parietal-occipital pair; (3) delta/alpha 1 left intra-hemispherical LLC in occipital-temporal pair; (4) theta/alpha 1 left intra-hemispherical LLC in occipital-temporal pair; (5) theta/alpha 1 right intra-hemispherical LLC in occipital-temporal pair; (6) theta/alpha 2 right intra-hemispherical LLC in occipital-temporal pair.

The following 15 EEG markers overcome the threshold of 70% stablished as “moderate” classification rate (see Table 7): (1) Delta/alpha 1 current density in central region; (2) Delta/alpha 1 current density in parietal region; (3) Delta/alpha 1 current density in occipital region; (4) Delta/alpha 1 current density in temporal region; (5) Delta/alpha 1 current density in limbic region; (6) Theta/alpha 1 current density in frontal region; (7) Delta/alpha 2 current density in central region; (8) Delta/alpha 2 current density in parietal region; (9) Delta/alpha 2 current density in occipital region; (10) Delta/alpha 2 current density in temporal region; (11) Delta/alpha 2 current density in limbic region; (12) Delta/alpha 1 inter-hemispherical LLC in occipital region; (13) Theta/alpha 1 left intra-hemispherical LLC in occipital-temporal pair; (14) Alpha 1 right intra-hemispherical LLC in parietal-limbic pair; (15) Theta/alpha 1 right intra-hemispherical LLC in occipital-temporal pair.

**Table 7:** **Results of the classification between single AD and Nold subjects based on EEG markers of source activity and lagged linear connectivity**.

**LORETA sources**	**Sensitivity (%)**	**Specificity (%)**	**Accuracy (%)**	**AUROC**
Central delta/alpha 1	71.7	71	71.4	0.73
Parietal delta/alpha 1	74.2	73	73.6	0.79
Occipital delta/alpha 1	73.3	78	75.5	0.82
Temporal delta/alpha 1	78.3	70	74.5	0.78
Limbic delta/alpha 1	75.8	72	74.	0.77
Frontal theta/alpha 1	0	72	70.9	0.76
Central delta/alpha 2	65.8	70	67.7	0.71
Parietal delta/alpha 2	68.3	76	71.8	0.77
Occipital delta/alpha 2	71.7	75	73.2	0.79
Temporal delta/alpha 2	63.3	78	70	0.76
Limbic delta/alpha 2	66.7	74	70	0.73
Occipital delta/alpha 2 inter-hemispherical LLC	65.8	66	65.9	0.70
Occipital-temporal theta/alpha 1 left intra-hemispherical LLC	66.7	65	65.6	0.70
Parietal-limbic alpha 1 right intra-hemispherical LLC	65	68	66.4	0.70
Occipital-temporal theta/alpha 1 right intra-hemispherical LLC	70.8	72	71.4	0.74

*The classification rate is computed by the analysis of area under the receiver operating characteristic curve (AUROC). The table reports the classification indexes for all the EEG markers having an AUROC higher than 0.70 (i.e., 70%)*.

**Figure 6:**
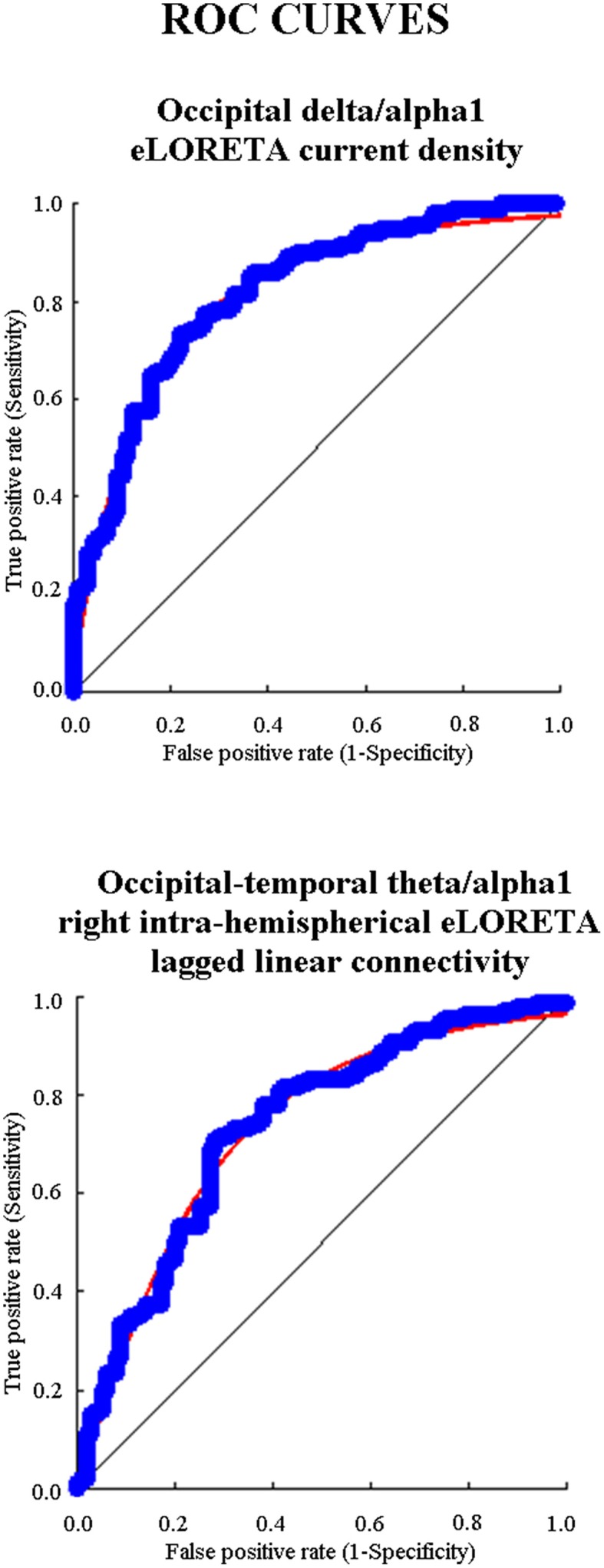
**Diagram showing the ROC (receiver operating characteristic) curves that illustrate the performance of the EEG markers with the best classification rate of single Nold and AD individuals**. The upper panel shows the best EEG marker for source activity and the bottom panel the best EEG maker for functional connectivity. These EEG markers had an area under the ROC curve (AUROC) higher than 0.70 (i.e., 70%), which is the threshold of a moderate classification performance.

Among these EEG markers, delta/alpha 1 current density in occipital region reached the following best classification rate: the specificity of 78%, the sensitivity of 73.3%, the accuracy of 75.5%, and AUROC of 82%. Regarding the EEG markers of LLC, theta/alpha 1 right intra-hemispherical LLC in occipital-temporal pair reached the best classification rate: the specificity of 72%, the sensitivity of 70.8%, the accuracy of 71.4%, and AUROC of 74%. Figure 6 shows the ROC curves for these both composite EEG markers.

### Results of the fourth statistical session: Relationship between the EEG markers and relevant variables of AD

The MMSE score as an index of global cognition was on average 19.9 (± 0.5 SD) in the AD-subgroup (*n* = 53) and 18.2 (± 0.4 SD) in the AD+ subgroup (*n* = 67). Independent *t*-test indicated that the difference of the MMSE score between the two AD subgroups was statistically significant (*p* < 0.001), in line with the working hypothesis.

**Figure 7:**
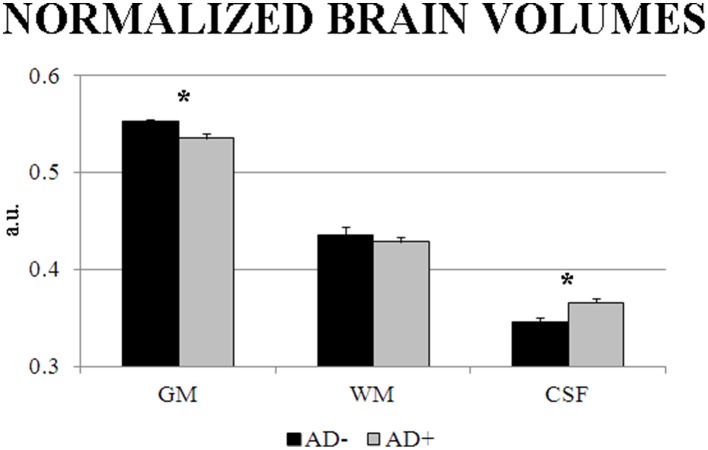
**Mean and *SD*-values of cortical gray matter (GM), subcortical white matter (WM), and cerebrospinal fluid (CSF) normalized volumes as indexes of brain structural integrity extracted by magnetic resonance imaging (MRI) in a subpopulation of 39 AD patients having MRI data associated to EEG recordings**. The values are reported in the AD subgroup negative to the EEG marker (AD−; *n* = 12) and in the AD subgroup positive to the EEG marker (AD+; *n* = 27). These values refer to an ANOVA design showing a statistically significant interaction [*F*_(2, 74)_ = 3.3761, *p* < 0.05] between the factors Group (AD−, AD+) and Volume (GM, WM, CSF). Asterisks indicate the *p* level of the statistical differences between the two AD subgroups obtained by Duncan *post-hoc* testing.

Table 8 reports information about personal and clinical characteristics of the AD− and AD+ individuals of 39 AD patients having MRI associated with EEG recordings. Concerning the MRI markers of structural brain integrity, the GM, WM, and CSF normalized volumes of the AD− subgroup (*n* = 12) were on average 0.55 (±0.003 Standard Error—SE), 0.44 (±0.009 SE), and 0.35 (±0.005 SE), respectively. Furthermore, they were 0.54 (±0.004 SE), 0.43 (±0.006 SE), and 0.37 (±0.005 SE), respectively, in the AD+ subgroup (*N* = 27). The ANOVA design showed a statistically significant interaction [*F*_(2, 74)_ = 3.3761, *p* < 0.05] between the factors Group (AD− subgroup, AD+ subgroup; independent variable) and Volume (GM, WM, CSF). Duncan *post-hoc* testing indicated that the GM normalized volume was lower in the AD+ subgroup compared to the AD− subgroup (*p* < 0.05), while the CSF normalized volume was higher in the AD+ subgroup compared to the AD− subgroup (*p* < 0.001; see Figure 7). Instead, the between-groups difference in the WM volume did not reach the statistical significance (*p* > 0.05).

**Table 8:** **Demographic and clinical data of the subgroups of AD patients (AD− and AD+) in the subpopulation of 39 AD patients having MRI data associated to EEG recordings**.

	**Gender (Female/Male)**	**Age (years)**	**Education (years)**	**MMSE (score)**
AD− (*n* = 12)	10/2	73.4 ± 1.7 SE	7.8 ± 1.7 SE	19.8 ± 1.3 SE
AD+ (*n* = 27)	16/11	66.9 ± 1.5 SE	8.5 ± 1.0 SE	17 ± 0.6 SE

*MMSE, Mini Mental State Evaluation*.

## Discussion

Our “BRAINON” research program has been testing markers of resting state eyes-closed EEG rhythms to characterize neurophysiological correlates of AD at group level (Babiloni et al., 2004a,b, 2008, 2009a, 2010, 2011a,b,c, 2013a,b,c). These markers were extracted by LORETA/eLORETA, popular software aimed at estimating power and connectivity of cortical sources of EEG rhythms (Pascual-Marqui et al., 1994, 2007a). Results at group level showed abnormalities of these EEG markers especially at delta and alpha frequency bands (Babiloni et al., 2004a,b, 2008, 2009a, 2010, 2011a,b,c, 2013a,b,c). In the present study, we moved from group to the individual level. We tested the ability of some of these spectral EEG markers to classify single Nold subjects and AD patients with dementia, as a preliminary step toward their use for a neurophysiological assessment of AD patients.

A preliminary control analysis of the present study confirmed previous evidence of our research group (Babiloni et al., 2004a,b, 2008, 2009a, 2010, 2011a,b,c, 2013a,b,c). Concerning the cortical sources of the EEG power, main results showed a higher activity of the posterior delta sources together with lower activity of the alpha sources in the AD group compared to the Nold group. To integrate this information content, we produced composite EEG markers by computing the ratio between the activity of posterior delta and alpha sources in relevant cortical regions.

Concerning the functional connectivity of the EEG sources, the present results displayed a lower intra- and inter-hemispherical connectivity at alpha rhythms in the AD group compared to the Nold group. Of note, the use of eLORETA allowed overcoming the methodological limitations due to the use of standard FFT to compute the bivariate spectral coherence between electrode pairs. With respect to multivariate estimators like eLORETA, bivariate measures of EEG functional interrelatedness may produce spurious connections due to the common feeding effect (i.e., false coupling between sources B and C due to the common influence from a source A) and head volume conduction effects (Blinowska and Kaminski, 2013; Kaminski and Blinowska, 2014).

As a novelty, the present ROC curve analysis indicated that some EEG markers of intra- and inter-hemispherical source connectivity at low-frequency alpha had an AUROC higher than 70%, which is the threshold of a moderate classification rate (DeLong et al., 1988). Among them, the best classification rate was reached by the functional connectivity between right occipital and temporal sources in the ratio between low-frequency alpha and theta: the sensitivity of 70.8%, the specificity of 72%, the accuracy of 71.4%, and AUROC of 74%.

In the same line, the ROC curve analysis showed that some EEG markers of cortical sources of EEG power had an AUROC higher than 70%. They mainly comprised the ratio between low-frequency alpha and delta source activity in posterior cortical regions. Among them, the best classification rate was reached by the ratio between occipital low-frequency alpha and delta source activity: the sensitivity of 73.3%, the specificity of 78%, the accuracy of 75.5%, and AUROC of 82%.

Overall, these present results pointed to a moderate classification rate of EEG markers of source power and functional connectivity for the detection of single AD individuals. In precedence, several studies tested various spectral EEG markers for the classification of the Nold and AD individuals. They reported classification rates between 82 and 90% using advanced mathematical classifiers including the ANNs (Anderer et al., 1994; Pritchard et al., 1994; Adler et al., 2003; Brassen et al., 2004; Lehmann et al., 2007; Dauwels et al., 2009).

### Clinical significance of the EEG topographic markers of AD

What is the potential clinical significance of the present EEG markers? According to the new guidelines of 2014 (Dubois et al., 2014), these EEG markers should not be used to formulate a diagnosis of AD. Indeed, they do not directly reflect the pathophysiological markers of AD such as Aβ1-42 and tau in the cerebrospinal fluid or brain. Rather, they should be considered as candidate EEG topographic markers for the assessment of the neurophysiological mechanisms underlying the fluctuation of cortical arousal and vigilance in the relaxed wakefulness. Indeed, these neurophysiological mechanisms generate cortical delta and alpha rhythms in healthy humans, which derange in AD patients (Babiloni et al., 2015).

The present results suggest that EEG markers of source power and functional connectivity in relaxed wakefulness may enrich the neurophysiological assessment of AD patients with dementia, although the best EEG marker for the classification between Nold and AD individuals was the occipital sources of delta/low frequency alpha. This best EEG marker may be useful to differentiate the status of brain function in AD patients with similar values in MMSE, autonomy in the daily life, and cognitive reserve as indexed by education years and intellectual occupations across lifespan (Snowdon, 2003; Riley et al., 2005; Tyas et al., 2007). Cognition, daily life abilities, and cognitive reserve status being equal, an AD patient with an abnormality of the EEG marker (e.g., one standard deviation beyond the mean value of a reference Nold population) may reflect more neurophysiological “frailty” and less brain reserve than an AD patient negative for this EEG marker. In this example, the AD patient with neurophysiological “frailty” should receive more therapeutic resources and clinical attention. This clinical perspective is supported by the present evidence showing that compared to the AD patients with normal EEG marker, those with an abnormal EEG marker (e.g., one standard deviation beyond the mean value of a reference Nold population) did exhibit lower MMSE score, lower normalized cortical gray matter density, and greater normalized cerebrospinal fluid, as signs of an impairment of global cognition and brain structural integrity, respectively.

### Methodological remarks

Compared to the mentioned classification rates of the previous studies, the moderate classification rate of this study was reasonably due to some intrinsic methodological limitations. First, our large clinical and EEG dataset (i.e., 120 AD vs. 100 Nold) was acquired by several clinical units, thus increasing the variance of the data due to a possible slightly different local implementation of clinical exclusion/inclusion criteria and EEG procedures. Second, no AD patient with severe dementia was enrolled in the present study (they typically show highly abnormal delta and alpha rhythms, which inflate classification rate). Third, all enrolled AD patients were under treatment by standard long-term Acetylcholinesterase inhibitors, which are expected to preserve alpha rhythms and reduce the difference between Nold and AD subjects in this respect (Brassen and Adler, 2003; Geldmacher, 2003; Onofrj et al., 2003; Babiloni et al., 2006d). Fourth, some of the enrolled Nold subjects (chosen among AD relatives or caregivers) might indeed suffer from a preclinical stage of AD, thus confounding the classification task. In fact, they were diagnosed based on traditional clinical criteria (e.g., NINCDS-ADRDA, McKhann et al., 1984; DSM-IV), without an assessment of the pathophysiological markers of AD (Dubois et al., 2014). Of note, we mitigated this risk including only Nold subjects having 27 or higher score of MMSE.

## Conclusions

In our previous studies, spectral EEG markers of relaxed wakefulness were found to be abnormal in groups of AD patients with dementia (Babiloni et al., 2004a, 2006a, 2009b, 2011a,b, 2013b). Can these EEG markers be used for the classification of single Nold and AD individuals, as a premise for future clinical applications? The results of the present study showed that 15 spectral EEG markers of source power and functional connectivity had a classification rate higher than 70%, computed by the analysis of ROC curves. Most of them were based on the ratio between alpha and delta source power in posterior cortical regions. The best classification rate was reached by the ratio between occipital low-frequency alpha and delta source activity. It exhibited the following performance: the sensitivity of 73.3%, the specificity of 78%, the accuracy of 75.5%, and AUROC of 82%. These results pointed to a moderate classification rate of several EEG markers of source power and functional connectivity for the detection of single AD individuals. Future studies will have to test the classification rate of these relevant EEG markers as a multivariate input to advanced mathematical classifiers such as the ANNs.

## Author contributions

CB, RF, AT, RL, CP, and VB gave their contributions to the conception and design of the work. AS, RF, FN, LG, RT, VC, MB, AG, PS, SA, GB, GS, GL, and GF were responsible for the enrollments, all the clinical evaluations, and the recordings of EEG data. AT, RL, SC, JM, AB, and CP performed the analysis of data. CP, RL, GT, and VB performed the statistical analysis. CB, AT, RL, and CP gave their contribution in the interpretation of the results. CB, AT, RL, and CP contributed in drafting the work and revising it critically for important intellectual content. AS, RF, FN, LG, GL, GF, and AB contributed in the revision of the first and later versions of the manuscript.

### Conflict of interest statement

The authors declare that the research was conducted in the absence of any commercial or financial relationships that could be construed as a potential conflict of interest.
